# Clinical and Radiological Outcomes of Treating Closed Humeral Shaft Fractures in Adults Using a Functional Brace

**DOI:** 10.7759/cureus.89837

**Published:** 2025-08-11

**Authors:** Mohammed Redwan, Amanda Almouslem, Zain Habib, Mohammed Arifuzaman, Yasser Iskandar

**Affiliations:** 1 Trauma and Orthopaedics, North Manchester General Hospital, Manchester, GBR; 2 Hospital Medicine, Damascus University, Damascus, SYR; 3 Trauma and Orthopaedics, Manchester University NHS Foundation Trust, Manchester, GBR; 4 General Surgery, North Manchester General Hospital, Manchester, GBR; 5 Trauma and Orthopaedics, Damascus University, Damascus, SYR

**Keywords:** bracing, conservative approach, functional bracing, humeral fracture, humeral shaft fracture

## Abstract

Introduction: This was a prospective case series to evaluate the clinical and radiological outcomes of treating closed humeral shaft fractures in adults using a functional brace.

Methods: Twelve adult patients (mean age: 36 years; range: 16-70 years) with closed humeral shaft fractures meeting the inclusion criteria were initially managed with a U-shaped slab for two weeks, followed by a functional brace until radiological union was achieved. Clinical and radiological assessments were conducted weekly during the first month post-bracing, and biweekly thereafter until union. Clinical outcomes were evaluated using Hunter’s criteria. Shoulder and elbow functions were assessed using the Constant-Murley Score (CMS) and Mayo Elbow Performance Score (MEPS), respectively. The shoulder and elbow range of motion (ROM) was measured at final follow-up. Radiological alignment was assessed in the coronal and sagittal planes at initial presentation and at the end of treatment.

Results: All 12 fractures (100%) achieved union at a mean of 10 weeks (range: 7-12 weeks). According to Hunter’s criteria, eight patients (66.7%) had excellent outcomes (Grade V), and three patients (25%) had very good outcomes (Grade IV). The mean CMS was 91.8, with nine (75%) patients scoring above 90. The mean MEPS was 96.2. Final angular displacement averaged 5.6° in the coronal plane and 1.5° in the sagittal plane. Spontaneous correction of displacement was observed without manipulation. No significant correlation was found between final angular displacement and functional outcomes (p > 0.05).

Conclusion: Functional bracing for closed humeral shaft fractures in adults results in a high union rate and excellent clinical and radiological outcomes. It is a safe and effective treatment option for this injury.

## Introduction

Humeral shaft fractures are relatively common, accounting for approximately 3% of all fractures, and pose a significant challenge to orthopedic surgeons [1]. These injuries can lead to a substantial reduction in individual productivity and negatively impact work performance [2]. A humeral shaft fracture is defined as a break occurring distal to the surgical neck and proximal to the humeral condyles. There is an ongoing debate regarding the most appropriate treatment method, as a variety of conservative and surgical options exist.

The incidence of humeral shaft fractures shows a bimodal distribution: a smaller peak (25 per 100,000) occurs in adolescents due to high-energy trauma, while a larger peak (100 per 100,000) is seen in older adults, particularly women aged 60-80 years, typically resulting from low-energy mechanisms [3].

Diagnosis of humeral shaft fractures

Clinically, diagnosis is usually straightforward following a clear traumatic event. Patients often present with severe pain, visible limb deformity, ecchymosis, abnormal mobility, and crepitus at the fracture site. A complete neurovascular assessment of the affected limb is essential.

Radiologically, diagnosis is confirmed with anteroposterior and lateral X-rays, ensuring the images include both shoulder and elbow joints [4].

Treatment of humeral shaft fractures

The treatment of humeral shaft fractures has evolved significantly since its documentation by the ancient Egyptians in 1600 BC [5]. Despite advancements, the fundamental goals of treatment remain unchanged: to achieve complete fracture union with proper alignment and restore full limb function. Treatment options range from non-operative (conservative) approaches to various surgical interventions, chosen based on patient factors (age, comorbidities) and fracture characteristics (pattern, displacement, soft tissue condition).

Conservative management remains the mainstay for most humeral shaft fractures, achieving union rates up to 90% with favourable clinical outcomes [1]. Among the conservative techniques, the functional brace has emerged as the preferred option due to its simplicity, adjustability, allowance for early joint movement, and low cost [6].

Surgical treatment - including plating, intramedullary nailing, and external fixation - is typically reserved for specific cases, such as open fractures, polytrauma, failed conservative treatment, or situations where bracing is not tolerated. While surgical methods can provide excellent outcomes, they carry inherent risks such as iatrogenic radial nerve injury, extended hospitalisation, and increased healthcare costs [7].

Several anatomical and biomechanical characteristics of the humerus influence treatment decisions; the humerus is the most mobile long bone in the body, with additional movement from scapular motion, increasing the likelihood of malunion in multiple planes. It functions as a lever in the upper limb, experiencing significant angular forces but relatively little compressive load. When a person is upright, the humerus hangs vertically under gravity, which can assist with fracture alignment. The bone is well-covered by soft tissue and has a rich blood supply, supporting robust healing.

Acceptable parameters for non-surgical union include the following: (1) anterior-posterior angulation (< 20°), (2) varus/valgus angulation (< 30°), (3) rotational deformity (< 30°), and (4) shortening (< 3 cm).

These factors support the assertion made by John Charnley that the humerus is the easiest long bone to treat conservatively [8].

While conservative treatment may not achieve perfect anatomical reduction, this is often unnecessary due to the compensatory range of motion of the shoulder and elbow. Historically, various methods have been used, including hanging casts, U-splints, shoulder spicas, and olecranon pin traction. However, these methods often restrict early joint mobility, leading to stiffness [6].

In 1977, Sarmiento introduced the functional brace for treating humeral shaft fractures, allowing early movement of the shoulder and elbow while promoting fracture alignment through soft tissue compression [9]. The brace consists of anterior and posterior shells contoured to the arm and connected by adjustable straps. Its mechanism of action involves compression of surrounding soft tissues, creating hydrostatic pressure and muscle contraction that assist in realigning fracture segments. Gravity also aids in the reduction process [6].

During the initial week of treatment, an arm sling may be used for comfort. As pain subsides, patients are encouraged to begin active movement of the shoulder and elbow. Once swelling decreases, usually within 7-14 days, the initial immobilisation is replaced with the functional brace, which is worn until clinical and radiological signs of union are observed [9]. The pressure applied by the brace should be adjusted as oedema resolves, and attention must be given to skin care beneath the brace to prevent irritation or infection.

This modern strategy provides a dynamic form of stabilisation that encourages early mobilisation and takes advantage of the body’s natural healing mechanisms. It has proven to be an effective and safe approach for the management of most closed humeral shaft fractures [6].

Despite the widespread use of functional bracing for closed humeral shaft fractures, there remains limited prospective data evaluating both clinical and radiological outcomes in a well-defined patient population. Furthermore, existing literature often lacks detailed functional assessments using validated scoring systems. This study addresses these gaps by prospectively evaluating outcomes in adult patients treated with a standardised functional bracing protocol.

The primary objective of this study was to assess the time to radiological union and clinical outcomes using Hunter’s criteria. The secondary objectives included evaluating shoulder and elbow function using the Constant-Murley Score (CMS) and Mayo Elbow Performance Score (MEPS), measuring final range of motion (ROM), and assessing fracture alignment in the coronal and sagittal planes at union.

## Materials and methods

This prospective study was conducted between April 1, 2021, and March 31, 2022. Adult patients (aged ≥ 16 years) presenting to the emergency department with closed humeral shaft fractures treated conservatively were eligible for inclusion. Follow-up continued until September 30, 2022. Institutional review board approval was not sought for this study as it was an observational review of standard care without any deviation from established clinical practice.** **

Initial evaluation was performed by the on-call orthopaedic team and included a thorough neurovascular examination of the affected limb. Anteroposterior and lateral radiographs of the humerus were obtained to assess fracture characteristics. Displacement was measured in both the coronal (varus/valgus) and sagittal (anteroposterior) planes. Patients were selected for conservative management based on the eligibility criteria detailed in Table 1.

**Table 1:** Eligibility criteria for conservative treatment Eligibility criteria for conservative treatment [6,9].

Parameter	Acceptable limit
Anteroposterior angulation	Less than 20°
Coronal plane angulation	Less than 30° (Varus/Valgus)
Rotational deformity	Less than 30°
Shortening	Less than 3cm

Initial immobilisation was achieved using a U-shaped slab, with the elbow positioned at 90° flexion and the forearm in a neutral position, supported with an arm sling. After approximately two weeks, once oedema and acute pain had subsided, a functional brace was applied. The brace was sized according to the contralateral limb and allowed anterior elbow exposure and flexion up to 120°. Adhesive straps were progressively tightened to accommodate soft tissue changes and reduce movement at the fracture site.

Following brace application, patients continued to use an arm sling for an additional two weeks. Active and passive movements of all limb joints were encouraged during this time. Straps were adjusted as needed to maintain appropriate compression while accommodating limb volume reduction.

Radiographic and clinical evaluations were performed weekly for the first month following the application of the brace and biweekly thereafter until fracture union. The brace was removed once both radiological signs of healing and clinical indicators, such as the absence of pain and abnormal mobility, were present.

Delayed union was defined as the absence of radiographic and clinical healing within four to six months, while non-union was defined as failure to achieve union beyond six months.

At final follow-up, six months post-injury, patients underwent comprehensive clinical and radiological evaluation. Clinical outcomes were assessed using Hunter’s criteria, a qualitative grading system originally described by Hunter for evaluating the functional results of humeral shaft fracture treatment [10]. Patients were classified into the five grades of Hunter's criteria, based on fracture union, pain, ROM, and residual deformity, as listed in Table 2.

**Table 2:** Hunter's grading criteria Qualitative parameters for Hunters Grading [10].

Hunter's Grade	Qualitative Parameters
Grade I (Poor)	Non-union or severe functional impairment
Grade II (Fair)	Delayed union or moderate limitation of motion and/or deformity
Grade III (Good)	Union with mild functional limitation or residual angulation
Grade IV (Very Good)	Union with minimal deformity and full or near-full function
Grade V (Excellent)	Complete union, full and pain-free range of motion, no deformity

Shoulder function and ROM were assessed using the CMS [11], while elbow function was evaluated using the MEPS [12]. Final radiographic analysis included angular deformity measurements in the coronal and sagittal planes.

All data were collected and analysed using Statistical Product and Service Solutions (SPSS, version 22; IBM SPSS Statistics for Windows, Armonk, NY) and Microsoft Excel (Microsoft® Corp., Redmond, WA). Descriptive statistics were used to summarise the data, including means, standard deviations, and proportions where appropriate. For inferential analysis, statistical tests such as the chi-squared test for categorical variables and independent samples t-tests (or non-parametric equivalents where assumptions were not met) for continuous variables were employed. A p-value of <0.05 was considered statistically significant, and all results were reported primarily in terms of p-values to indicate the strength of associations or differences observed.

## Results

A total of 26 adult patients (age > 16 years) presented to the emergency department with closed humeral shaft fractures during the study period. Of these, 11 patients were excluded due to surgical management; eight due to fracture irreducibility, one polytrauma case, one pathological fracture, and one with associated neurological injury. Three additional patients were excluded due to loss to follow-up. The final study sample comprised 12 patients.

The cohort included six males and six females, with a mean age of 36.1 years (range: 16-70 years). Fractures occurred on the left side in seven patients (58.3%) and on the right in five (41.7%). Fracture location was proximal third in four patients (33.3%), middle third in seven (58.3%), and distal third in one patient (8.4%). Most fractures were classified as AO type A (nine cases, 75%), with type A3.2 (simple oblique fractures of the midshaft) being the most common subtype (four cases, 33.3%).

Low-energy trauma, falls from standing height, was the predominant mechanism of injury (six patients, 50%), followed by motor vehicle collisions (four patients, 33.3%) and work or sports injuries (two patients, 16.7%).

Initial radiographic alignment

Most fractures showed varying degrees of displacement. In the coronal plane, varus angulation was the most frequent deformity. One fracture (8.3%) was undisplaced, four (33.4%) had mild varus angulation (1°-10°), six (50%) were moderately displaced (11°-20°), and one fracture (8.3%) had a severe varus deformity of 30°. The mean initial varus displacement was 13.8°.

In the sagittal plane, four fractures (33.3%) were undisplaced, six (50%) had mild posterior angulation (1°-10°), and two (16.7%) had moderate posterior angulation (11°-20°). The mean posterior angulation was 5.5°.

Fracture healing and complications

All fractures achieved union (100%) without surgical intervention. The mean time to radiological union was 10 weeks (range: 7-12 weeks). Two patients (16.7%) developed brace-related dermatitis, managed conservatively without interruption of treatment.

Functional outcomes

At final follow-up, shoulder flexion and internal rotation were preserved in all patients. Shoulder abduction was full in seven patients (58.3%), mildly limited (by 10°) in four patients (33.3%), and moderately limited (by 20°) in one patient (8.3%). The mean shoulder abduction was 135°. External rotation was mildly restricted (10° loss) in two patients (16.7%); the remainder had full range. Elbow ROM (flexion-extension) was normal in all patients. No patient demonstrated instability or limitation in elbow function.

**Figure 1:**
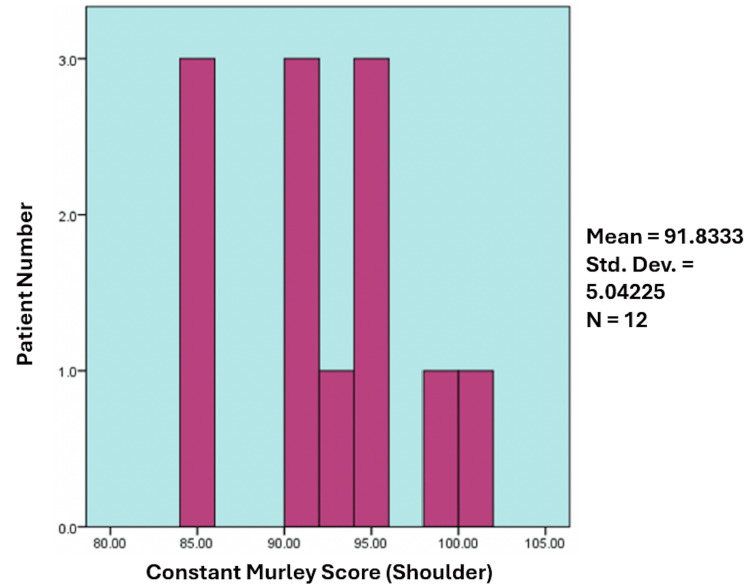
Sample distribution according to the Constant-Murley Shoulder Score (CMS)

The mean CMS was 91.8 (range: 85-100), with nine patients (75%) achieving excellent scores (>90) and three patients (25%) classified as good (range: 80-90). This is depicted below in Figure 1. Despite favourable scores, a statistically significant difference was noted compared to the normal contralateral limb (p < 0.005). The mean MEPS was 96.2 (range: 90-100), indicating excellent outcomes in all patients, with no significant difference compared to the unaffected side (p > 0.05).

A paired t-test comparing CMS between the affected and contralateral limbs revealed a statistically significant difference (mean difference: 2.7 ± 3.0; t = 3.11; df = 11; p < 0.005), despite the affected side demonstrating high functional scores (mean CMS: 91.8 ± 5.0). This suggests a small but measurable reduction in shoulder function compared to the unaffected limb.

In contrast, the MEPS showed no statistically significant difference between the affected and contralateral limbs (mean MEPS: 96.2 ± 3.0; p > 0.05), indicating excellent and symmetric elbow function in all patients post-treatment.

According to Hunter’s criteria, eight patients (66.7%) had excellent outcomes (Grade V), three (25%) had very good outcomes (Grade IV), and one patient (8.3%) had a good outcome (Grade III). The latter improved clinically following physiotherapy and was reclassified as Grade IV. This is depicted in Table 3.

**Table 3:** Distribution of patients according to Hunter’s grade after treatment Hunter's grading criteria: Grade V (Excellent): Complete union, full and pain-free range of motion, no deformity. Grade IV (Very Good): Union with minimal deformity and full or near-full function. Grade III (Good): Union with mild functional limitation or residual angulation. Grade II (Fair): Delayed union or moderate limitation of motion and/or deformity. Grade I (Poor): Non-union or severe functional impairment

Hunter's Grade V	Hunter's Grade IV	Hunter's Grade III	Hunter's Grade II	Hunter's Grade I
8 Patients	3 Patients	1 Patient	0	0
66.7%	25%	8.3%	00%	00%

Final radiographic alignment

At union, most fractures showed minimal residual displacement. In the coronal plane, three fractures (25%) were undisplaced, eight (66.7%) had minor varus displacement (1°-5°), and one fracture (8.3%) had 15° of varus angulation. The mean final varus displacement was 5.6° (range: 0°-15°).

In the sagittal plane, nine fractures (75%) were undisplaced, and three (25%) had minor posterior angulation (range: 1°-10°). The mean posterior displacement was 1.5° (range: 0°-8°). This distribution of fracture displacement in the coronal and sagittal planes is depicted in Tables 4-5. No significant correlation was observed between final angular displacement and clinical outcome in either plane (p > 0.05).

**Table 4:** Distribution of fractures according to coronal displacement on the final assessment

Total	Nondisplaced	Mild Varus Displacement (1-5°)	Moderate Varus Displacement (>15°)
12	3 Fractures	8 Fractures	1
%	25%	66.7%	8.3%

**Table 5:** Distribution of fractures according to sagittal displacement on the final assessment

Total	Nondisplaced	Mild Posterior Displacement (1-10°)	Moderate Posterior Displacement (20-11°)
12	9 Fractures	3 Fractures	0
%	75%	25%	0%

Chi-square analysis summary

Chi-square tests were conducted to assess the statistical significance of categorical distributions. A significant deviation from a uniform distribution was found in Hunter’s score grading (χ²(2) = 6.50, p = 0.039, Cramér’s V = 0.52), indicating a strong preference toward excellent outcomes. External rotation stiffness also showed statistical significance (χ²(1) = 6.00, p = 0.014, V = 0.71), reflecting a large effect size. Shoulder abduction stiffness demonstrated a non-significant trend (χ²(2) = 4.50, p = 0.105, V = 0.43). No significant associations were observed between fracture side and outcome (χ²(1) = 0.33, p = 0.564, V = 0.17), nor between gender and outcome (χ²(2) = 1.33, p = 0.514, V = 0.30). Final sagittal axis displacement was not significantly associated with outcome (χ²(1) = 3.00, p = 0.083, V = 0.50).

These findings suggest that, despite radiographic deformity, conservative treatment with functional bracing yielded consistently favourable clinical outcomes, with minimal loss of function and low complication rates.

## Discussion

The present study evaluated the clinical and radiological outcomes of treating closed humeral shaft fractures in adults using a functional brace. Our findings demonstrate a 100% union rate, excellent functional outcomes, and a low complication profile, supporting the continued use of non-operative management in appropriately selected cases.

Functional bracing was introduced by Sarmiento et al. [6] in the 1970s and has since been widely adopted for the treatment of closed diaphyseal humeral fractures [8,13]. Its primary advantage lies in the allowance of early mobilization while maintaining adequate alignment through soft tissue compression. In our series, all fractures united without the need for surgical intervention, with an average union time of 10 weeks, which is consistent with previously reported ranges of 8-12 weeks [14,15].

The high SMS and MEPS in our cohort indicate excellent functional recovery. Shoulder abduction was mildly restricted in five patients but did not significantly affect daily function. These findings align with studies by Ali et al. and Zagorski et al., which reported minimal residual stiffness in the shoulder and elbow following functional bracing [15,16].

Radiologically, our results confirm the expected phenomenon of spontaneous correction of angular displacement over time, particularly in the sagittal plane. Although initial varus or posterior angulation was observed, the final mean deformity was minimal, with no statistically significant impact on function - supporting the notion that minor angulation does not necessarily compromise outcome [7,17]. Notably, our data did not show a significant correlation between the degree of residual deformity and final functional scores, a finding echoed by Sarmiento [9] and Denard et al. [17].

The complication rate was low, limited to two cases of dermatitis due to the brace. Importantly, there were no cases of non-union, malunion requiring correction, or nerve injury. These findings further validate the safety and efficacy of functional bracing, especially in resource-limited settings or when surgical risks outweigh potential benefits.

While surgical fixation may be preferred in certain situations - such as open fractures, polytrauma, or failed conservative management - our results reinforce that non-operative treatment remains a reliable first-line approach in the majority of closed, isolated humeral shaft fractures [18].

This study is limited by its small sample size and lack of a control group treated operatively. Additionally, while the prospective design and objective outcome measures enhance validity, longer follow-up is needed to assess long-term shoulder function and late complications.

## Conclusions

This prospective case series supports functional bracing as a safe and effective treatment for closed humeral shaft fractures in selected adult patients. While the outcomes were uniformly favourable in this small cohort, larger comparative studies are needed to confirm these findings and to identify patient or fracture characteristics that may influence treatment success.
